# Trauma-Informed Care: a Strategy to Improve Primary Healthcare Engagement for Persons with Criminal Justice System Involvement

**DOI:** 10.1007/s11606-018-4783-1

**Published:** 2019-03-25

**Authors:** Simran Chaudhri, Kimberly Caramanica Zweig, Preetha Hebbar, Sonia Angell, Ashwin Vasan

**Affiliations:** 1 Health Access Equity Unit, New York City Department of Health and Mental Hygiene, Long Island City, NY USA; 2 Division of Prevention and Primary Care, New York City Department of Health and Mental Hygiene, Long Island City, NY USA; 3 Division of General Medicine, Department of Medicine, Columbia University Irving Medical Center/NewYork-Presbyterian Hospital, New York City, NY USA; 4 Department of Population and Family Health, Mailman School of Public Health, Columbia University, New York City, NY USA

**Keywords:** trauma, trauma-informed care, criminal justice, patient engagement, quality of care, implementation, primary care

## Abstract

Trauma is pervasive in the USA, but disproportionately present in individuals and communities burdened by poverty, violence, and exposure to the criminal justice system. Engagement in clinical care, especially community-based primary care, is particularly important in the immediate period following community reentry from incarceration, where opportunities to engage clients in services are essential for improved health and reduced recidivism. Trauma-informed care offers an important and innovative opportunity for healthcare systems and primary care providers to improve quality of care and the patient experience, thereby increasing longitudinal engagement of marginalized and hard-to-reach patient populations like persons with criminal justice system exposure. Trauma-informed care implementation includes educating providers and transforming practices to incorporate safety, trust, peer support, collaboration, empowerment, and cultural perspectives into everyday operations and care delivery. While comprehensive trauma-informed care involves transformation on a system level, trauma-informed approaches can also be adopted by the individual provider to improve the clinical consultation. By recognizing the role of trauma and its impact on an individual’s physical, emotional, and behavioral health, providers and clients can build mutual trust, focus on individual growth, and begin to foster healing.

Michael, a 48-year-old man, came home 6 weeks ago after being in prison for 15 years. Michael has a personal history of childhood trauma and has never trusted anyone enough to talk about the violence he experienced at a young age or while incarcerated. He suffers from high blood pressure and diabetes and is worried about refilling his medications on his own. The medication supply he was given when released ran out, and he is waiting for his Medicaid to be reinstated. While he is aware of the dangers of his condition if left unmanaged, he does not want to see a doctor because he mistrusts authority, has had negative experiences with healthcare in the past and while incarcerated, and does not want to waste time in waiting rooms when he should be out looking for work. He is having difficulty finding stable housing and steady income, which has contributed to his depressed mood and frequent drinking, all of which have been exacerbated by the stress and stigma associated with reintegrating into the community after incarceration.

## WHY TRAUMA IS A SIGNIFICANT ISSUE FOR PERSONS WITH JUSTICE INVOLVEMENT

Individual trauma results from an event or series of events that are physically or emotionally harmful and have lasting adverse effects on mental, physical, social, and emotional well-being.^1^ In the USA, diagnosed and untreated trauma is a health and social epidemic; high prevalence of adverse childhood experiences (ACEs) has been reported in a number of populations and can range from 52^2^ to 68.2%.^3^ Trauma is pervasive across income and demographic groups, but is disproportionately present, and has a disproportionate impact, in individuals and communities burdened by poverty, violence, social isolation, racism, and exposure to the criminal justice system (CJS). Indeed, rates of lifetime exposure to trauma are elevated among persons with justice involvement (PWJI) compared to the general population,^4–7^ as a direct result of incarceration or via cumulative trauma across the life course in communities marked by structural racism and violence.^3, 8^ PWJI are defined as individuals who have had contact with the police or criminal court system, which can range from a series of brief interactions like being stopped, questioned, and frisked; being detained or incarcerated in jail or prison; or being under supervised release in the community via probation or parole. They are among the most medically and socially complex populations^9–11^ due to a unique combination of traumatic early life experiences, cumulative disadvantage in their communities and lived experiences,^12^ and circumstances faced when reentering the community after jail or prison.^13^

Trauma exposure among incarcerated persons has been associated with a range of behavioral health conditions, including alcohol and substance use and mental illness.^6, 7^ In general, exposure to traumatic life events in childhood and adulthood is associated with psychological and mood disorders, decreased physical health, increased risk-taking behaviors (such as tobacco use and unsafe sex), obesity and disordered eating, chronic diseases, and reduced engagement with healthcare services.^1, 14–16^ Further, for marginalized communities disproportionately exposed to the CJS, these adverse outcomes are often compounded by the trauma of everyday stigma, discrimination, and racism, which has been shown to increase the risk of stress, depression,^17^ hypertension,^18^ cardiovascular disease,^19^ specific cancers,^20^ and overall mortality.^21^

## WHY TRAUMA SHOULD BE ADDRESSED IN PRIMARY CARE

**Fig. 1:**
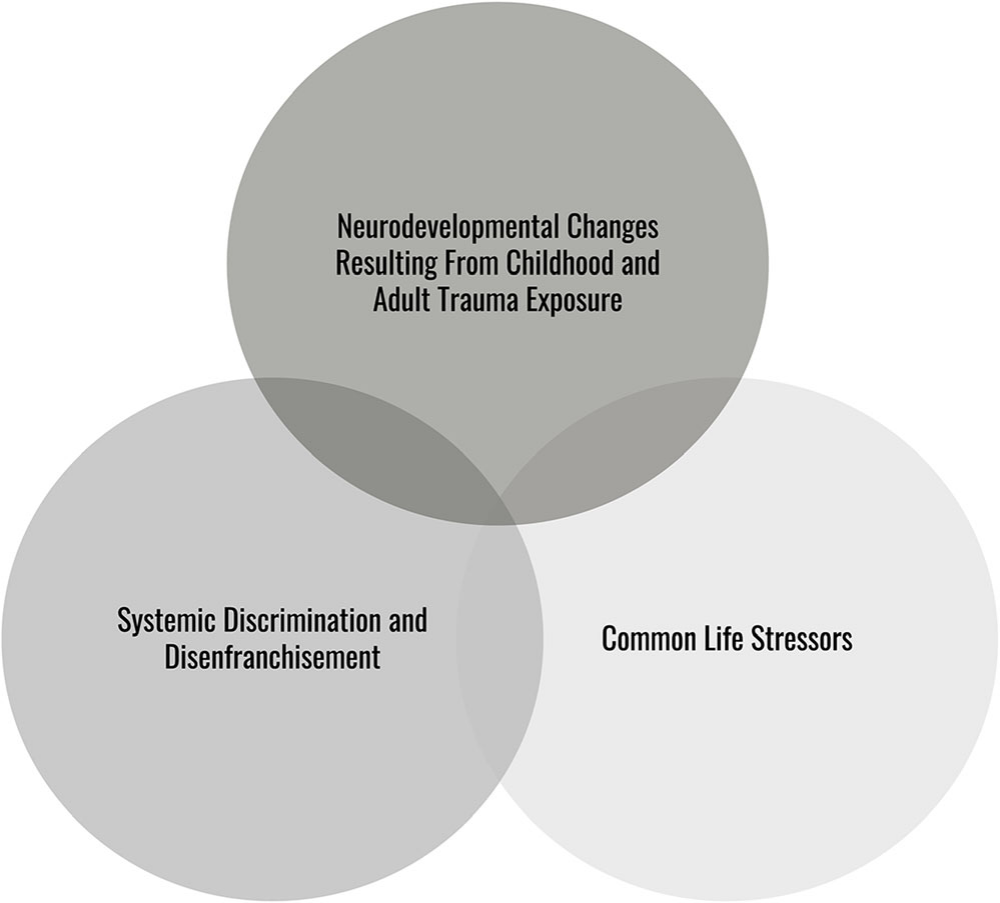
Upstream factors that impact healthcare-seeking behaviors and engagement for persons with criminal justice system involvement.

Despite the pervasiveness of trauma, especially among PWJI,^4–7^ it remains relatively unseen and unaddressed by healthcare systems, with the exception of pediatrics,^22, 23^ where universal ACE screening has become standard of care given pediatricians’ role as mandatory reporters. The failure of healthcare systems to systematically address the impacts of trauma across the life course not only affects the quality of care provided to traumatized populations, but can also negatively impact long-term patient engagement in health and social services. On the individual level, the complex interplay between trauma-related neurodevelopmental changes, systemic discrimination and disenfranchisement, and ongoing life stressors (Fig. 1) can result in maladaptive behaviors that impede healthy behaviors and healthcare engagement, including difficulties with communication, conflict management, and emotional regulation skills. Providers may also be subject to the impacts of vicarious trauma via their interactions with traumatized patients and can, in turn, exhibit their own maladaptive behaviors.^24^ Ultimately, if unaddressed, patient trauma can fragment the therapeutic alliance, reduce quality of care, impede long-term patient engagement, and contribute to provider burnout.^24–26^ For PWJI, the potentially re-traumatizing effects of interacting with healthcare services can be further compounded by perceptions of racism, classism, and criminal record discrimination by providers, which have been independently linked to low levels of engagement into health services.^27–29^ Instead of primary care, they often preferentially use relatively anonymized, transactional care from emergency departments and urgent care centers, limiting the potential for engagement, partnership, and follow-up.^28, 30, 31^

## ADDRESSING TRAUMA IN PRIMARY CARE: TRAUMA-INFORMED CARE

Trauma-informed care (TIC) offers primary care an important and innovative opportunity to improve the quality of care and the patient experience, thereby increasing engagement of PWJI and other marginalized groups into longitudinal care and community-based social services. Engagement is particularly important during the community reentry phase from incarceration, where the disproportionate burden of premature mortality occurs,^9^ and where opportunities to engage and retain clients into services are essential for improved health and reduced recidivism.^32^ Adapting community-based health and human services to more comprehensively meet the needs of trauma-exposed clients is a promising strategy that could increase patient health-seeking behavior and engagement, facilitating strong connections to patient-centered care that can address the health and social needs of PWJI.

TIC is defined by the Substance Abuse and Mental Health Services Administration (SAMHSA) as services and organizational cultures that:

1. *Realize* the widespread impact of trauma and understand potential paths for recovery;
2. *Recognize* the signs and symptoms of trauma in clients, families, staff, and others involved with the system;
3. *Respond* by fully integrating knowledge about trauma into policies, procedures, and practices;
4. Seek to actively resist *re-traumatization*.^1^

Implementing TIC includes educating providers and transforming practices to incorporate six guiding principles into everyday operations and care delivery^1^: safety^2^; trustworthiness and transparency^3^; peer support^4^; collaboration and mutuality^5^; empowerment, voice, and choice; and^6^ cultural, historical, and gender issues.^1^ In healthcare delivery, implementation of TIC in the clinical environment may involve programming within and across a number of domains, as detailed in Table 1, which can be adapted to each clinic’s unique setting, patient population, care environment, and operational needs.

**Table 1:** Trauma-Informed Programming in Different Healthcare Domains

Healthcare domain	Examples of trauma-informed programming	Examples of trauma-informed programming for a patient with justice involvement
Care environment	Create welcoming, easy to navigate spaces that minimize visual, auditory, or other potentially re-traumatizing triggers	Assess for and minimize things that can be triggering in the clinic environment, ex. uniformed security guards. Include reentry service organization pamphlets or posters to signal welcome and belonging
Dialog and interactions between patients, staff, and providers	Focus on positive, accepting language that facilitates patient safety, disclosure, and engagement, and create a supportive work environment for the entire care team	Recognize unfamiliarity with community healthcare systems and explain the reasoning behind common screening questions and what to expect during examinations and procedures. Always obtain consent before examination. Support patients to make choices and regain a sense of control over their bodies and healthcare
Patient and provider workflows	Reduce barriers to care access (e.g., insurance coverage, physical access to the clinic), and facilitate efficient and effective patient throughput, provider workflows, and meaningful patient-provider interactions	Medicaid is suspended or revoked while incarcerated and can take time to reinstate upon release; see patients whose Medicaid may not yet be reinstated as services can be billed retroactively
Standard operating procedures	Incorporate trauma-informed principles into all aspects of clinic operations, including human resources, budgeting and financial management, and infrastructure, including incorporating trauma survivors and those with lived experience (e.g., CJS involvement) as a part of the care team	Hire peers with experience of the criminal justice system as health educators or community health workers. Budget a small amount of funds to support recently released patients with immediate needs (a meal, clothing, bus passes, etc.), potentially increasing trust and engagement. Make walk-in appointments available to allow patients to see a clinician on their terms
Trauma screening and disclosure	While data are limited, some TIC advocates have called for upfront and universal trauma screening, including screening adults for ACEs, which can provide a better understanding of a patient’s trauma history, allow for targeted interventions, and encourage normalization and disclosure as an act of healing.^33^ Weigh the pros and cons of trauma screening before deciding if it is right for your clinic	Should you decide to screen for trauma or CJS involvement, ensure that staff are trained in proper screening techniques and appropriate support is available for patients, including behavioral health and social service referral options. In the absence of screening, all staff can be trained in patient-centered communication strategies and how to appropriately respond if a patient discloses CJS involvement
Self-regulation and social resilience	Build concrete behavioral modification tools for staff, providers, and clients to manage everyday states of emotional-hyperarousal, facilitate better communication, and strengthen relationships^22, 34^	When creating tools and guidance, study perspectives of healthcare inside correctional facilities and understand how these experiences can contribute to a patient’s emotional state and engagement in care when back in the community

## TRAUMA-INFORMED PRIMARY CARE: EARLY FINDINGS

There are some early, encouraging signs that TIC design and implementation is moving beyond pediatrics into other healthcare settings, with innovative models being piloted at various primary care locations nationwide, and SAMHSA currently drafting guidelines for TIC in primary care settings.^1, 33, 35^ Examples of trauma-informed primary care approaches currently being piloted include all staff training on the impact of trauma on general health and well-being,^35, 36^ universal ACEs screening for all children and adults,^37^ creating critical incident management teams and training staff in de-escalation techniques,^37^ engaging patients in organizational planning and program design,^36, 37^ creating a safe and welcoming environment that minimizes trauma triggers,^37, 38^ and preventing secondary traumatic stress in staff.^36–38^ Early research conducted in primary care settings has demonstrated that TIC significantly improves the patient–provider relationship,^39, 40^ and is a key contributor to overall healthcare quality and engagement into care.^41^ These promising TIC interventions are important in their own right, but become especially so on top of existing nationwide efforts to integrate behavioral health into primary care, comprehensively addressing a patient’s mental and physical well-being.

## LIMITATIONS AND NEXT STEPS

Barriers remain to implementing TIC in primary care, namely, the lack of a clear implementation model on which to design trauma-informed interventions. Specific challenges and roadblocks include transforming an organization’s established culture, policies, and practices; the limited evidence around trauma screening practices; and accessing behavioral health services for patients requiring referrals. Implementation science and program evaluations are required to elucidate evidence-based approaches to trauma-informed screening, consultation, and clinic transformation that can be disseminated throughout a variety of primary care settings. Further research is also required to determine the impact TIC has on engagement in primary care and preventative services, behavior change, health outcomes, and healthcare cost savings for PWJI.

Acknowledging an individual’s complex history is crucial to the provision of effective, patient-centered services and a strong therapeutic alliance. While we know that PWJI have disproportionate exposure to trauma that can hinder primary care engagement, they are clearly not the only population impacted by traumatic life experiences. As such, TIC remains an important tool as primary care attempts to improve engagement and quality of care for other marginalized populations including refugees, survivors of violence, people experiencing homelessness, and veterans, among others. By recognizing the role of trauma in an individual’s life and its impact on physical, emotional, and behavioral health, providers and clients together can build mutual trust, focus on individual growth and development, and begin to foster healing. At a systems level, TIC in healthcare has the potential to improve quality and increase engagement into primary care, thereby increasing timely and appropriate care utilization, improving health outcomes, and reducing costs.^42^ Through TIC, primary care has the ability to catalyze change, holistically and successfully supporting their clients with CJS involvement.

## WHAT A TRAUMA-INFORMED PRIMARY CARE VISIT CAN LOOK LIKE FOR A PERSON WITH JUSTICE INVOLVEMENT

Our patient, Michael, apprehensively walks into a primary care clinic for the first time in 20 years. He is greeted warmly by a receptionist who informs him of how long he can expect to wait and invites him to speak with her if he has any questions. Sitting down, he is worried about sharing his incarceration experience with his doctor but notices a pamphlet for a local reentry service organization that puts his mind somewhat at ease. After 30 min in a busy waiting room, and with growing anxiety about having to make his mandatory parole appointment later, he becomes agitated and begins pacing. The receptionist notices, and following the clinic’s trauma-informed protocol, offers him a seat in a quieter corner and finds his physician to explain the situation and flag him as someone who may require special attention. Michael is transferred to the consultation room, where a few moments later the physician knocks on the door to give him a warning before walking in; she sits down on his level, looks him in the eye, introduces herself, and asks what his goals are for the visit. Michael discloses his recent release and need to get his medications. Sensing his hesitation, she pauses to acknowledge how hard that experience must have been for him and positively reinforces his decision to tell her, explaining that her aim is to tailor care to meet all his needs and get him the best care possible. Before taking a clear history, she asks generally if he feels incarceration has impacted his health, allowing him to describe it in his own words. Recognizing his lack of familiarity with the healthcare system, she is sure to explain why she is asking any sensitive questions and what to expect throughout the consultation. She obtains consent before beginning the examination, explains her findings, and answers questions. She offers choices for referrals and follow-up and discusses their pros and cons, helping Michael to develop a sense of empowerment and ownership of his care, something that he was not privy to while incarcerated. While the physician recognizes that she cannot address all his complex needs in a single appointment, she prioritizes getting his medications organized and creating a safe and welcoming rapport so that he will come see her again.
